# Pellagra

**Published:** 1911-12

**Authors:** 


### Books and Magazines.

PELLAGRA. By Dr. A. Marie, Physician to the Asylums, Department of the Seine, Editor-in-Chief, Archives de Neurologie, and Director of the Laboratory of Pathological Psychology, Ecole des Hautes Etudies, Paris, France. With Introductory Notes by Professor Lombroso. Authorized Translation from the French by C. H. Lavinder, M. D., Past Assistant Surgeon, U. S. Public Health and Marine Hospital Service, and J. W. Babcock, M. D., Physician and Superintendent State Hospital for the Insane, Columbia, S. C. With additions, illustrations, bibliography and appendices. 1910. The State Co., Publishers, Columbia, S. C. Cloth, $2.50.

This is a most important and timely book, especially to the Texas profession. The recognition in our midst of this terrible disease is so recent, and it is so prevalent and so fatal, and so little is really known of it, that Dr. Marie's hand-book ought to and will meet a large clientele and a good sale. It gives in small space quite a comprehensive history of the disease, its symptoms, course and treatment, so far as they are known. The author adheres to Lombroso's dictum as to its etiology—diseased corn.

Pellagra is not confined to the poor, badly nourished and insanitary, as is generally supposed.

